# Designing biomaterials with immunomodulatory properties for tissue engineering and regenerative medicine

**DOI:** 10.1002/btm2.10063

**Published:** 2017-05-16

**Authors:** James I. Andorko, Christopher M. Jewell

**Affiliations:** ^1^ Fischell Department of Bioengineering University of Maryland College Park MD 20742; ^2^ Department of Microbiology and Immunology University of Maryland Medical School Baltimore MD 21201; ^3^ Marlene and Stewart Greenebaum Cancer Center Baltimore MD 21201; ^4^ United States Department of Veterans Affairs Baltimore MD 21201

**Keywords:** biomaterial, immunology, intrinsic immunogenicity, nanoparticle and microparticle, regenerative medicine, tissue engineering, vaccine and immunotherapy

## Abstract

Recent research in the vaccine and immunotherapy fields has revealed that biomaterials have the ability to activate immune pathways, even in the absence of other immune‐stimulating signals. Intriguingly, new studies reveal these responses are influenced by the physicochemical properties of the material. Nearly all of this work has been done in the vaccine and immunotherapy fields, but there is tremendous opportunity to apply this same knowledge to tissue engineering and regenerative medicine. This review discusses recent findings that reveal how material properties—size, shape, chemical functionality—impact immune response, and links these changes to emerging opportunities in tissue engineering and regenerative medicine. We begin by discussing what has been learned from studies conducted in the contexts of vaccines and immunotherapies. Next, research is highlighted that elucidates the properties of materials that polarize innate immune cells, including macrophages and dendritic cells, toward either inflammatory or wound healing phenotypes. We also discuss recent studies demonstrating that scaffolds used in tissue engineering applications can influence cells of the adaptive immune system—B and T cell lymphocytes—to promote regenerative tissue microenvironments. Through greater study of the intrinsic immunogenic features of implantable materials and scaffolds, new translational opportunities will arise to better control tissue engineering and regenerative medicine applications.

## 1 INTRODUCTION

Biomaterials have enabled advances in fields spanning tissue engineering, drug delivery, vaccination and immunotherapies, and implantable devices. This breadth is due to the ability of these materials to encapsulate and protect cargos (e.g., chemicals, cells, and proteins), to provide biocompatible supports (e.g., devices, scaffolds), and to allow facile modification of chemical and physicochemical properties.^1^, ^2^ Not surprisingly, biomaterials range from naturally occurring biological building blocks to fully synthetic substances. This ever‐expanding use of biomaterials is also creating increasing need for deeper understanding of the interactions between materials and the biological environments they encounter. Nowhere is this need more evident than the immune engineering field. Biomaterials are being widely explored in vaccines and immunotherapies to combat infectious disease, cancer, and autoimmunity, but the early clinical successes of these approaches are few and far between. One of the interesting findings in the field—described in seminal papers published less than a decade ago^3^, ^4^—is that many biomaterials exhibit intrinsic material properties that can activate immune pathways. This is certainly an opportunity to gain knowledge that informs design of materials that could actively bias immune responses toward desired functions. In tissue engineering, many emerging strategies are also employing immune cues and cells in biomaterial‐based structures for engineering organs and tissues, and for regenerative medicine. In contrast to vaccine and immunotherapy research, the immunological role that scaffolds or other materials might play—through intrinsic properties, modification of surface chemistry, or other tunable strategies—has yet to take center stage. Since essentially every tissue engineering application either interacts with or specifically seeks to avoid the immune system, understanding these roles could provide a new lever to improve tissue engineering and regenerative medicine.

**Figure 1:**
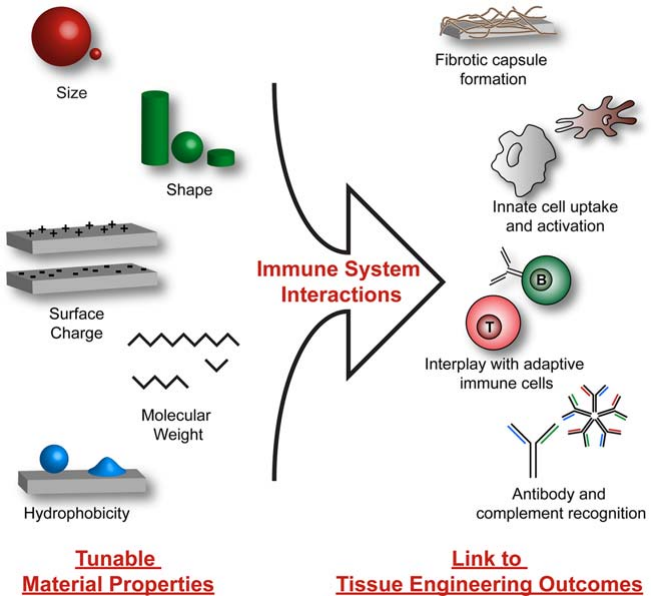
Intrinsic properties of materials influence immune responses. Biomaterials commonly used in vaccine, immunotherapy, and tissue engineering approaches exhibit features including size, shape, surface charge, hydrophobicity, and molecular weight that alter interactions with the immune system. Encountering components of the innate and adaptive immune system with these materials results in formation of fibrotic capsule to isolate the material, differential activation of dendritic cells and macrophages, recognition and removal by antibody and complement proteins, and even manipulating adaptive immune response

This review will discuss what has been learned about the role physicochemical properties of biomaterials play in directing immune responses from the vaccine and immunotherapy fields, and analyze how these concepts might be exploited for tissue engineering and regenerative medicine. We begin with a brief introduction to the immune system and the response to injury and implanted materials. Next, we discuss what is known about how immune response is impacted by biomaterial properties such as size, shape, and stability/molecular weight, along with surface features such as chemical functionality, charge, and hydrophobicity (Figure 1). Then, we describe how the introduction of biomaterial scaffolds, and the specific features of these tissue engineering constructs exhibit intrinsicly immunogenic features that can impact immune cell polarization and wound healing. Finally, we highlight new research directions leveraging the intrinsic properties of materials to control immune function and push the forefront of tissue engineering and regenerative medicine.

## 2 IMMUNE PATHWAYS ACTIVATED BY PATHOGENS AND OTHER FOREIGN MOLECULES ALSO RESPOND TO BIOMATERIALS

Across vaccines, immunotherapies, and tissue engineering, the immune system represents both an opportunity for and barriers against successful outcomes. To protect the body from harmful pathogens, the immune system has evolved over time to quickly recognize pathogens or other non‐self agents through general physicochemical features that are uncommon in the body. These include the particulate nature of bacteria and viruses, repetitive molecular patterns such as the polysaccharides that comprise bacterial cell walls, and structural motifs such as hydrophobic regions or double stranded RNA often found in molecules from viruses and bacteria.^5^, ^6^ One component of the innate immune system—the segment that provides rapid, but less specific immune responses—are pattern‐recognition receptors (PRRs). PRRs are present on antigen presenting cells (APCs), including dendritic cells (DCs) and macrophages, that scan the body for danger‐associated molecular patterns (DAMPs) and pathogen‐associated molecular patterns (PAMPs).^7^, ^8^ PRRs, including toll‐like receptors (TLRs) and the inflammasome, identify specific DAMPs and PAMPS and initiate different signaling pathways leading to the clearance of potentially harmful agents.^9^, ^10^ The inflammasome is a cytoplasmic complex of proteins which activates caspases and the release of IL‐1β, a key cytokine involved in initiating inflammatory processes.^9^, ^10^ Interestingly, the inflammasome has been associated with immune cell activation in response to treatment with adjuvants, materials commonly added to vaccine formulations to increase immunogenicity or potency. One ubiquitous example is alum, an FDA‐approved adjuvant consisting of particulate aluminum salt formulations.^9^, ^10^, ^11^ Since many existing vaccines use alum, and numerous technologies involving microparticles and nanoparticles are in development as vaccines and immunotherapies, continued research into how these materials activate the inflammasome is of great interest. The complement system is another mechanism of the immune system, consisting of serum proteins that assemble after encountering microbes or other extracellular pathogens. These proteins form complexes that tag, destroy, and eliminate the invading pathogens.^7^, ^12^ Supporting these fast‐acting innate immune responses is the adaptive immune system. In contrast to innate immunity, adaptive immunity arises more slowly, but is more specific and can lead to the generation of immunological memory. Broadly speaking, adaptive immunity includes cell‐mediated and antibody‐mediated responses against specific, foreign molecules, terms antigens.^7^ Cell‐mediated immunity involves activation of T lymphocytes that differentiation into cytotoxic T cells able to destroy self‐cells infected with intracellular pathogens such as viruses. Antibody‐mediated immunity arises from the activation of B lymphocytes that, upon differentiation, secrete antibodies that bind extracellular pathogens (e.g., bacteria) or toxins to neutralize and clear these targets.

The immune system, particularly innate immune cells, also plays a large role in the response to injury and after implantation of biomaterials.^7^, ^13^, ^14^, ^15^, ^16^, ^17^, ^18^ Initially, neutrophils and other innate immune cells infiltrate the site of injury to clear pathogens associated with the injury, and secrete cytokines and chemokines that recruit other immune cells (e.g., macrophages).^7^, ^15^, ^16^ The first macrophages to arrive at the injury site exhibit an inflammatory phenotype known as M1. These M1 macrophages phagocytose pathogens and damaged cells, and produce pro‐inflammatory factors such as inducible nitric oxide synthase (iNOS), TNF‐α, and IL‐12. These factors promote inflammation and removal of pathogens, and recruit lymphocytes involved in generating adaptive immune repsonses.^16^, ^17^, ^18^, ^19^, ^20^, ^21^, ^22^ M1 macrophages typically persist at the wound or implant site for 2–3 days after injury, at which time the function of these cells shifts toward the M2 macrophage phenotype; this phenotype is crucial for tissue repair and generating new blood vessels.^16^, ^17^, ^18^, ^19^, ^20^, ^21^, ^22^ M2 macrophages are functionally different from M1 macrophages. This phenotype exhibits increased expression of key genes involved in wound‐repair (e.g., arginase and Fizz1), secretes cytokines and growth factors to stimulate cell proliferation, and deposits extracellular matrix to support tissue regeneration.^7^, ^15^, ^16^, ^17^, ^18^ Macrophages also influence the response to implants by forming specialized foreign body cells. These cells create a fibrotic capsule around implanted materials that isolate the material from the surrounding environment of the body.^15^ Since M1 and M2 macrophages have different functions, there is an opportunity to target these cells as immunomodulatory players to direct responses to tissue engineering constructs. Currently, it is understood that the phenotype and activation of macrophages is not always a binary process but rather a spectrum where cells upregulate or downregulate specific markers as they transition from one form (pro‐inflammatory) to another (wound‐healing).^18^, ^19^, ^20^, ^21^, ^22^ Since implantation of biomaterials involves these same mechanisms, there is a need to understand how the properties of materials alter immune interactions in the context of tissue engineering.

Many biomaterials exhibit structural features that trigger recognition as DAMPs and PAMPs. In particular, the immune system commonly responds to the repetitive patterns of polymer chains that can resemble bacterial polysaccharides, hydrophobic portions of materials, and the particulate nature of microparticles and nanoparticles that share characteristic dimensions of bacterial and viral pathogens.^23^, ^24^, ^25^ Over the past decade, many studies have revealed that even the most common biomaterials can activate immune or inflammatory pathways in the absence of other immunostimulatory signals, and that the physicochemical material properties can alter the magnitude or features of this response. For example, DCs incubated on thin biomaterial‐based films of naturally derived polymers (e.g., alginate, agarose, chitosan, and hyaluronic acid) or synthetic polymers (e.g., polylactic‐co‐glycolic acid (PLGA)) induce differential expression of surface activation markers. These signals include DC maturation markers (e.g., CD40), major histocompatibility class II (MHCII) complexes—proteins responsible for presenting antigens to naïve B and T cells, and co‐stimulatory markers (e.g., CD80, CD86). These co‐stimulatory signals act as a second signaling cue necessary for activation of B and T cells in response to antigen presented in MHC complexes.^7^, ^26^ In other studies, particulate systems of PLGA, polystyrene, or silica were treated in conjunction with an inflammatory signal (e.g., TLR agonists) and the DC response to these treatments was evaluated.^3^, ^4^, ^27^ As expected, the inflammatory signal activated DCs, but interestingly, when treated along with particles, the particles synergistically increased the activation of DCs relative to the TLR agonist alone or the polymers alone. The pro‐inflammatory responses were also associated with inflammasome signaling at levels comparable to those measured by alum, the strong human adjuvant discussed above.^3^, ^4^, ^27^ These revelations have prompted a new area of study to understand which physicochemical properties influence intrinsic immune response and how these interactions occur. New studies are also now exploring such intrinsic immune features of materials to direct vaccine responses more specifically, to improve cancer therapies, and to promote immune tolerance in combating autoimmune diseases.^28^, ^29^, ^30^, ^31^, ^32^, ^33^, ^34^, ^35^, ^36^, ^37^, ^38^

## 3 UNDERSTANDING INTRINSIC IMMUNOGENICITY OF BIOMATERIAL DELIVERY SYSTEMS COULD INFORM TISSUE ENGINEERING APPLICATIONS

In the past few years the field has learned a great deal about how the properties of biomaterial vaccine and immunotherapy carriers trigger innate immune pathways. Many studies, recently reviewed,^39^, ^40^, ^41^, ^42^, ^43^, ^44^, ^45^, ^46^ seek to overcome or evade immunological barriers by modulating these properties of biomaterials‐based carriers. This is valuable insight that could be leveraged as the tissue engineering and regenerative medicine fields move forward. In this section, we will explore which properties of vaccine and immunotherapy carriers can be altered to modulate the immune response.

### 3.1 The size of biomaterial carriers alters uptake and APC activation

To drive immune responses, vaccines must reach lymph nodes, tissues that coordinate adaptive immunity through interactions between antigen‐experienced APCs and naïve B and T cells. Many vaccines rely on particle size or drainage through the lymphatics to provide a pathway from peripheral injection sites to the lymph nodes.^47^, ^48^, ^49^, ^50^, ^51^ Past studies have shown particles with diameters between 20 and 50 nm can passively drain through lymphatics, and larger particles are more dependent on phagocytosis by APCs which carry the engulfed cargo to lymph nodes.^47^, ^48^ The phenomena occur because particle trafficking is driven in part by the pressure gradient that exists between the blood and lymphatic vessels, causing convective forces that propel smaller particles into lymphatic vessels.^47^, ^48^, ^49^ As particle size increases, particles flow at much slower rates due to steric hindrance in the interstitial space, or mechanical size limitation for micron‐size particles. These larger particles are thus more reliant on internalization and trafficking by APCs to reach lymph nodes.^47^, ^48^, ^49^ Thus, the size of particles used to deliver vaccines and immunotherapies plays a large role in the uptake, trafficking, and retention of the cargo and carrier in immune cells and tissues.^52^, ^53^, ^54^

While particle size impacts trafficking and can alter how much carrier, drug, or vaccine reaches lymph nodes, changes in particle size also play a major role in modulating immune response. For example, with the same antigens and adjuvants present in a material‐based vaccine, the size of these particles biases the interaction with innate immune cells and skews the type of antibodies produced.^55^, ^56^, ^57^ In one recent study, gold nanoparticles with diameters of 3 and 12 nm were incubated with human‐derived DCs. These sizes caused different levels of DC activation, as indicated by common surface maturation markers (e.g., CD80, CD83, CD86, and MHCII) and inflammatory cytokine secretion.^58^ While both sizes of particles activated DCs, 3 nm particles caused higher activation levels and greater secretion of IL‐12 and IFN‐γ. These effects translated to increased pro‐inflammatory T cell function during co‐culture.^58^ In contrast, treatments with the 12 nm particles increased IL‐4 production, skewing the resulting T cell‐mediated immune responses toward a different function, wound healing.^58^ In another study, macrophages treated with particles of varying sizes induced different effects on the levels of the anti‐inflammatory cytokine IL‐10 and the pro‐inflammatory cytokine TNF‐α.^59^ While the largest particles did not induce cytokines, smaller particles with diameters ranging from 2 to 40 µm induced size‐dependent production of IL‐10 and TNF‐α; this activation was also shown to involve TLR‐2 stimulation.^59^ Many tissue engineering approaches involve implantation of macroscopic scaffolds or devices that degrade, release fragments, or experience wear effects.^15^ Thus, particulates can be generated that also trigger the same size‐dependent modulatory immune pathways.^15^ Additionally, bulk wear or fracture of implantable devices or scaffolds is often a key design focus since this can lead to acute device failure. From an immunological perspective, however, the inflammatory profile of the nano‐ and micro‐scale wear products or particles will take on an emerging roll in tailoring immune responses to improve device or implant performance.

### 3.2 Immune activation is influenced by biomaterial shape

While size is an important feature in determining trafficking, uptake, and intrinsic immunogenicity, the shape of materials also impacts these responses.^57^, ^60^, ^61^, ^62^, ^63^ For example, in one study, gold nanorods were internalized by macrophages at greater levels than nanospheres owing to preferential uptake of the former via micropinocytosis.^64^ In a separate study, glass rods were used as a tool to assess shape dependence by incubating rods of varying lengths with macrophages.^65^ Short rods were more rapidly taken up than longer rods, but interestingly, the longer rods, while not readily phagocytosed, induced greater levels of the inflammatory signals IL‐1α and TNF‐α.^65^ This inflammatory response was attributed to “frustrated” phagocytic interactions, a phenomenon that occurs when cells are unable to phagocytose larger‐scale objects. This failure results in the production of reactive oxygen species and inflammatory cytokines which ultimately could lead to chronic inflammation and fibrosis.^65^ This shape effect might also be important in the context of scaffolds and implants since these constructs are commonly too large for engulfment and often shed long fibers or other geometries upon degradation or wear. Other work has studied shape effects using titanium dioxide to prepare particles with diameters of 7–10 nm (anatase) or 15–20 nm (rutile), or nanotubes with diameters of 10–15 nm and lengths of 70–150 nm.^66^ These studies revealed shape dependence across cytokine secretion, production of reactive‐oxygen species, and DC maturation.^66^ In particular, the nanotubes generally caused the largest immunogenic effects,^66^ further demonstrating that particle shape impacts immunogenicity. Later in this section, we will discuss some of the mechanistic studies beginning to ascertain how the interactions of differently shaped particles with immune cells cause these differential effects.

**Figure 2:**
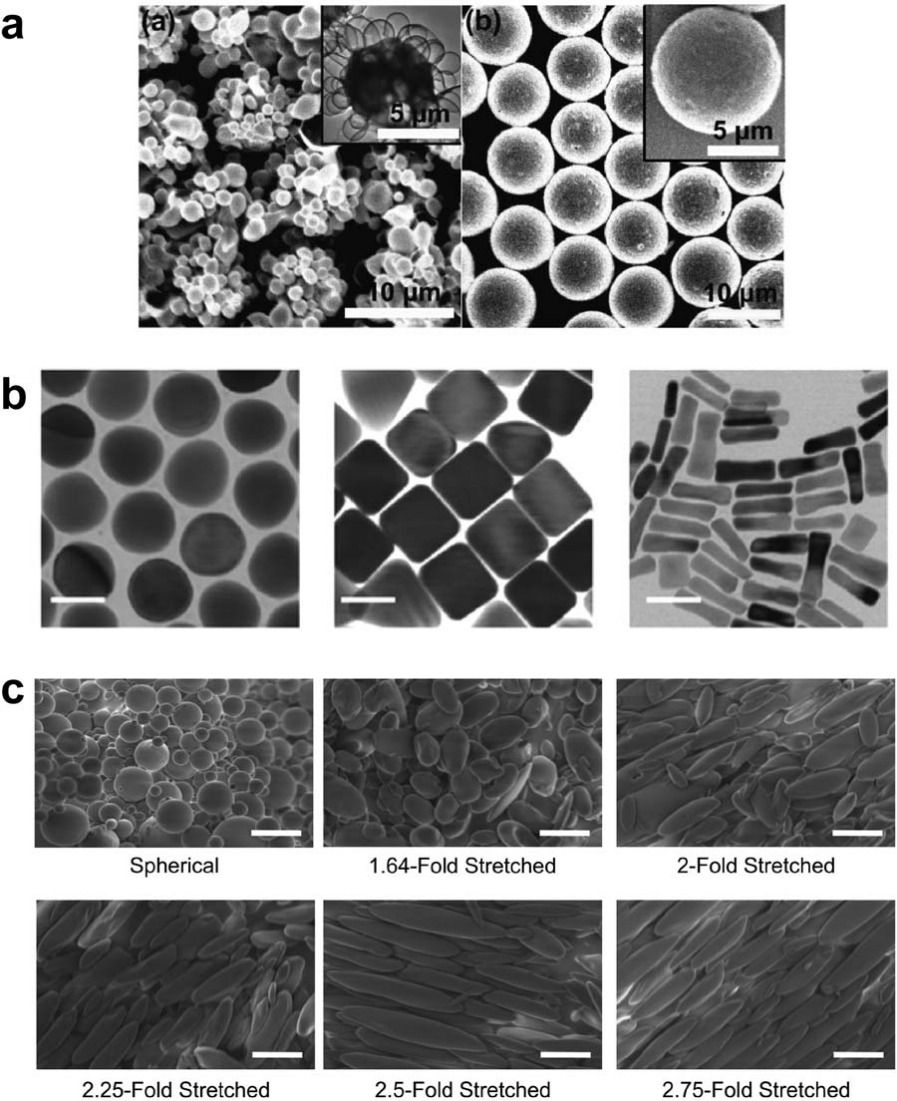
Particle shape dictates immune cell uptake and activation. (a) Spherical polymeric particles fabricated from polystyrene‐polyethylene oxide that exhibit rough surfaces (left) were preferentially taken up by macrophages and induced a pro‐inflammatory response compared to smooth particles (right) (Scale bar, 10 µm; inset scale bar, 5 µm). (b) Electron micrographs of gold nanoconstructs with spherical (left), cube (center), or rod‐like (right) shapes. When incubated with DCs, rod‐like particles induced inflammatory IL‐1β and activated the inflammasome, while sphere and cubes caused secretion of TNF‐α (Scale bar, 40 nm). (c) Spherical PLGA particles that are mechanically stretched to form ellipsoidal particles increase surface interactions with immune cells, leading to increased T cell proliferation. (Scale bar, 10 µm). (a) Reprinted with permission from Vaine CA, Patel MK, Zhu J, et al. Tuning innate immune activation by surface texturing of polymer microparticles: the role of shape in inflammasome activation. *J Immunol*. 2013;190(7):3525‐3532. (b) Reprinted with permission from Niikura K, Matsunaga T, Suzuki T, et al. Gold nanoparticles as a vaccine platform: influence of size and shape on immunological responses in vitro and in vivo. *ACS Nano*. 2013;7(5):3926‐3938. (c) Reprinted from Sunshine JC, Perica K, Schneck JP, Green JJ. Particle shape dependence of CD8+ T cell activation by artificial antigen presenting cells. Biomaterials. 2014;35(1):269‐277 with permission from Elsevier

The extent to which the shape of particulate carriers impacts inflammasome activation is important because, while seminal work has shown particle‐based carrier systems can act as adjuvants through inflammasome signaling, other work demonstrates these outcomes are not a feature of all particulate systems.^3^, ^4^, ^11^, ^27^, ^67^, ^68^ In a study from Vaine et al., particles synthesized from block copolymers to either exhibit rough or smooth surfaces (Figure 2a) differentially activated the inflammasome in mice.^69^ In this study, polystyrene‐polyethylene oxide particles with a rough surface morphology increased neutrophil recruitment and IL‐1β secretion compared with smooth polystyrene‐polyethylene oxide particles.^69^ This study also found that while the particles had comparable diameters, rough particles were preferentially taken up by macrophages, leading to increased inflammasome activity that was comparable to a positive control treatment with alum. Interestingly, this activation was absent when uptake was inhibited, suggesting that phagocytosis and endosomal destabilization were needed for the materials to activate the inflammasome.^27^, ^69^ In another study, antigen‐coated gold nanostructures formed as spheres, rods, or cubes (Figure 2b) triggered differential levels of cytokine secretion in DCs, leading to differences in antibody production in mice.^70^ The rod‐shaped particles induced an IL‐1β (i.e., inflammasome‐mediated) response, while spheres and cubes activated less specific (e.g., TNF‐α) inflammatory responses.^70^ These studies reveal that the inflammasome, an important pro‐inflammatory signaling cascade of the innate immune system, can be manipulated simply by altering particle shape. More work is needed to understand why these effects occur with some material shapes and not others. In particular, tissue engineering uses materials spanning biological building blocks to synthetic polymers, enabling a variety of shapes and topographies. Thus, understand how these different parameters promote or diminish inflammasome activation could allow more tunable constructs.

Research from the Green and Schneck groups has focused on using polymeric particles as artificial antigen presenting cells (aAPCs), mimicking the ability of APCs such as DCs to simultaneously present antigen fragments and costimulatory markers to T cells.^71^, ^72^, ^73^ In this work, the authors studied the impact of aAPC shape on T cell response.^71^, ^72^, ^73^ Spherical PLGA nanoparticles were first synthesized, then mechanically stretched to form elongated, ellipsoidal particles with different aspect ratios (Figure 2c). To properly mimic the way natural APCs interact with T cells, both antigen and costimulatory molecules need to be presented in the correct contexts to promote T cell activation and proliferation. To accomplish this, antigen within MHC molecules along with a co‐stimulatory antibody (anti‐CD28) were coupled to the surface of either spherical or ellipsoidal particles to form the biomimetic aAPCs.^71^, ^72^, ^73^ Since aAPCs need to interact with T cells, the parameters to ensure they remain extracellular were explored by incubating both spherical and ellipsoidal particles with macrophages and human umbilical cord vein endothelial cells. These studies revealed spherical aAPCs were phagocytosed quicker and at higher levels compared to ellipsoidal aAPCs.^72^ As a result, ellipsoidal aAPCs injected intravenously in mice experienced increased circulation time and greater biodistribution compared to spherical formulations. Since ellipsoidal aAPCs with increased circulation time had more opportunity to interact with T cells, this shape ultimately led to increased T cell proliferation compared to spherical aAPCs.^72^ Further analysis of aAPC properties revealed that the degree of stretching influenced the extent of T cell proliferation, with optimal stimulation occurring when aAPCs were stretched 2–2.5 fold, relative to the original diameter.^71^, ^72^ One possible reason for this difference is that the ellipsoidal aAPCs allowed increased contact length with T cells, supporting increased numbers of surface‐to‐surface interactions.^71^ Ultimately, treatment with ellipsoidal aAPCs displaying cancer antigens, in conjunction with a common cancer immunotherapy, reduced tumor burden and increased survival.^73^ The examples in this section demonstrate that micro‐ and nano‐scale shape changes can significantly alter the interactions with immune cells, promoting either a pro‐inflammatory or pro‐regenerative niche. These phenomena need to be further explored with tissue engineering constructs which exhibiting complex conformations, commonly employed to recapitulate the natural tissue the implant is replacing while allowing for multiple contact points with the cellular environment.

### 3.3 Biomaterial surface features and chemical functionality impact immune recognition

**Figure 3:**
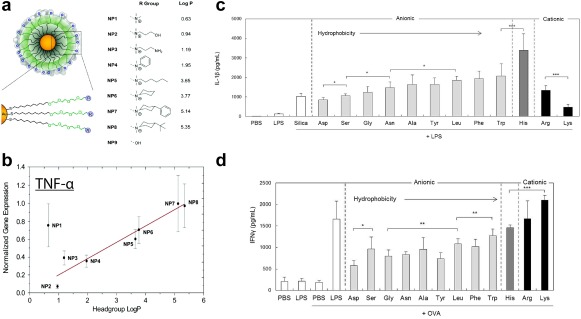
Surface chemistry of particulate systems impacts immunogenicity. (a) Gold nanoparticles are functionalized with different chemical (“R”) groups to exhibit varied hydrophobicity, denoted as “Log P.” (b) Immune cells isolated from mouse spleens were treated with these particles and revealed a correlation between increased hydrophobicity and elevated gene expression of pro‐inflammatory cytokines (TNF‐α). (c) Silica particles functionalized with different polypeptides with defined charges and levels of hydrophobicity increase the IL‐1β response to treatment with an immune stimulant (LPS). Increasingly hydrophobic surface chemistries promote increased IL‐1β secretion. (d) When these silica particles were treated in conjunction with a model antigen (OVA), particle immunogenicity increased T cell production of pro‐inflammatory IFN‐γ, with cationic particles inducing the highest levels. (a and b) Adapted with permission from Moyano DF, Goldsmith M, Solfiell DJ, et al. Nanoparticle hydrophobicity dictates immune response. *J Am Chem Soc*. 2012;134(9):3965‐3967. Copyright 2012 American Chemical Society. (c–d) Reprinted from Kakizawa Y, Seok Lee J, Bell B, Fahmy TM. Precise manipulation of biophysical particle parameters enables control of proinflammatory cytokine production in presence of TLR 3 and 4 ligands. *Acta Biomater*. 2017 with permission from Elsevier

As alluded to in Section 3.2, upon injection or implantation, surface features (e.g., roughness) and specific chemical moieties or properties can impact both the extent of interactions with immune cells and the immunogenicity.^74^, ^75^, ^76^, ^77^, ^78^ An important aspect along these lines is the role hydrophobicity plays in intrinsic immunogenicity.^23^, ^79^ The immune system has evolved to recognize molecules with highly hydrophobic portions as foreign, potentially dangerous materials (see Section 2). This property can thus trigger PRRs, leading to elimination.^23^ In one study, gold nanoparticles functionalized with increasingly hydrophobic chemical groups (Figure 3a) were incubated with immune cells isolated from the spleens of mice.^79^ Particles with greater hydrophobicity increased gene expression of pro‐inflammatory cytokines (e.g., TNF‐α, IFN‐γ) and similar effects were observed after intravenous injection in mice (Figure 3b).^79^ To combat the immunogenic effects of hydrophobic surfaces, hydrophilic molecules such as polyethylene oxide (PEO) and polyethylene glycol (PEG) are often added to delivery vehicles and tissue engineering scaffolds to increase hydrophilicity and reduce surface protein absorption.^80^, ^81^, ^82^ This increased hydrophilicity and resistance to protein absorption can also lead to decreased interactions with immune cells, which might reduce immunomodulatory responses.^80^, ^81^, ^82^ While diminishing interactions with immune cells could be beneficial in combating undesirable, pro‐inflammatory responses, future approaches could leverage changes in surface chemistry to bias immune response toward natural healing responses to injury.

The surface charge of a biomaterial also plays an important role in modulating immune function.^64^, ^68^, ^83^, ^84^ For example, using the same gold nanorod platform discussed in Section 3.2,^64^ surface charge of the nanoconstructs was found to impact immunogenicity, in addition to size and shape. Nanorods altered the inflammatory profile of macrophages, as indicated by changes in gene expression and surface activation markers, depending on the exposed functional groups.^64^ Amine‐terminated nanorods exhibiting a positive surface charge shifted macrophages to an anti‐inflammatory M2 phenotype, while carboxylic acid‐terminated nanorods with a negative surface charge induced an inflammatory M1 phenotype.^64^ Interestingly, other studies have revealed particles with positively charged surfaces lead to activation of the inflammasome at greater levels than negatively charged particles.^68^ Further, other work has shown particles with a negative surface charge can actually block or inhibit immune function.^84^, ^85^ In one such study, particles synthesized from carboxylated PLGA, polystyrene, or microdiamonds, all exhbiting negative surface charges, were able to supress inflammatory macrophages.^85^ These cells were the drivers of various mouse models of disease, including West Nile Virus brain infection, kidney injury, colitis, multiple sclerosis, and cardiac infarction. In each case, suppression was achieved following treatment with negatively charged particles by shifting macrophage accumulation from sites of disease to the spleen, where apoptosis of these cells occurred, leading to reduced inflammatory responses and promoting regulatory T cell phenotypes.^85^ As discussed directly in Section 3.4, the studies already presented reveal one of main challenges to understand intrinsic immunogencity, the difficulty in decoupling related properties such as hydrophobicity and chemical functionality.

Several studies have focused directly on the role of chemical functionality presented on surfaces to decipher how immue response is altered.^86^, ^87^, ^88^ For instance, peptides synthesized with either an L or D stereochemistry have been linked to a model antigen (ovalbumin, OVA) and used to vaccinate mice.^86^ The D stereochemistry, which is less susceptible to enzymatic degradation, led to stronger antibody responses and prolonged antigen presentation compared to the L stereotype.^86^ Additionally, it was determined that when macrophages were treated with particles that contained either oxidized, reduced, or native protein antigens, the particles with native or reduced antigen were preferentially taken up in vitro, and promoted strong immune responses in mice.^87^ The Fahmy Lab has used silica particles as a platform to understand how surface modification with polypeptides exhibiting different hydrophobicities and charges influence inflammasome signaling and DC activation in vitro.^88^ As expected based on the discussion above in Sections 2 and 3.1, these studies determined inflammasome activation was size dependent.^88^ Interestingly, however, investigation into how the surface chemistry of 300 nm particles impacted IL‐1β secretion revealed that particles functionalized with amino acids of increasing hydrophobicity drove increasing IL‐1β secretion (Figure 3c).^88^ Anionic particles caused the highest levels of activation, while cationic particles, shown above to activate pro‐inflammatory responses, induced lower levels of IL‐1β in this case. These differences were found to result from endocytic uptake and lysosomal rupture, a result supported by a reduction in IL‐1β secretion when uptake was chemically inhibited.^88^ When cells were treated with these particles mixed with OVA, cationic particles caused increased IFN‐γ production by T cells. Additionally, these elevated expression levels correlated to increasing particle hydrophobicity (Figure 3d).^88^ Since the balance of M1 and M2 macrophages plays a major role in many tissues engineering applications, the studies in this section highlight opportunities to modulate immune cell phenotypes by altering the hydrophobicity, charge, or surface chemical functional groups of materials used to fabricate tissue engineering constructs. Two common, naturally derived biomaterials, alginate and hyaluronic acid, are highly studied as scaffold materials and both exhibit negatively charged surfaces. The studies above have revealed that a negatively charged surface could either halt or promote immune responses. These differences highlight the need for more detailed studies to elucidate how hyaluronic acid, alginate, or other materials can be formulated to leverage surface charge in supporting regenerative outcomes.

### 3.4 Molecular weight and extent of material degradation impact immunogenicity

Many drug delivery and tissue engineering approaches employ biodegradable materials as vehicles or scaffolds to deliver signals to target cells or tissues over time. However, most of the studies investigating the intrinsic immunogenicity of materials have focused on a single snapshot in time or stage of degradation. Thus, there is a strong need to understand how the immunogenicity of materials change over time as degradation progresses, material properties change, and byproducts are formed.

Early research investigating how the degradation of biomaterials influenced immunogenicity centered on hyaluronic acid, an extracellular matrix glycosaminoglycan.^89^, ^90^, ^91^, ^92^ In this work, hyaluronic acid was enzymatically degraded to form fragments of varying molecular weight, then incubated with DCs or in DC and T cell co‐cultures. These experiments revealed low molecular weight hyaluronic acid (1500–5300 Da) increased DC activation, inflammatory cytokine secretion, and T cell proliferation by triggering TLR‐2 and TLR‐4 signaling.^89^, ^90^, ^91^, ^92^ Subsequent research has studied how macrophage differentiation is impacted by hyaluronic acid fragments with different molecular weights.^93^, ^94^ In these studies, macrophages were polarized to an M1 phenotype by low molecular weight hyaluronic acid, while high molecular weight hyaluronic acid induced a M2 phenotype, preferred for tissue repair and healing.^93^, ^94^

**Figure 4:**
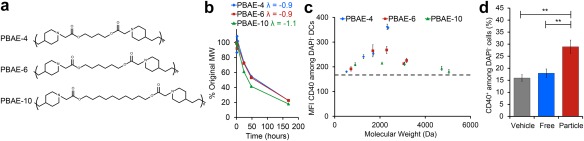
Polymer degradation and molecular weight influence DC activation in cell culture and mice. (a) PBAEs formulated with 4 (PBAE‐4, blue), 6 (PBAE‐6, red), or 10 (PBAE‐10, green) carbons in the diacrylate monomer backbone were synthesized with (b) different starting molecular weights, but similar degradation profiles. (c) PBAEs were degraded to distinct molecular weight ranges, formulated into particles, and used to treat DCs. Maximum activation, as indicated by CD40 expression on live (DAPI^−^) DCs, correlated with a molecular weight range of 1,500–3,000 Da. (d) After introduction into mice, immunogenic PBAE particles (red) induced statistically significant activation of lymph node resident immune cells compared to treatment with soluble (blue, “Free”) or buffer control (gray, “Vehicle”) treatments. (a–c) adapted with permission from Andorko JI, Pineault KG, Jewell CM. Impact of molecular weight on the intrinsic immunogenic activity of poly(beta amino esters). *J Biomed Mater Res A*. 2017;105(4):1219‐1229. (d) Reprinted with permission from Andorko JI, Hess KL, Pineault KG, Jewell CM. Intrinsic immunogenicity of rapidly‐degradable polymers evolves during degradation. *Acta Biomater*. 2016;32:24‐34

Our lab has investigated how the immunogenicity of synthetic polymers evolves as degradation progresses. Poly(beta amino esters) (PBAEs) provide an ideal system to address this question since these polymers are rapidly degradable and can be readily synthesized with different functionalities in a high‐throughput manner.^95^, ^96^, ^97^, ^98^ In one study investigating the effect of PBAE formulation and degradation on immunogenicity, PBAEs were formulated via Michael‐addition reactions between a diamine and one of three diacrylate monomers differing in the number of carbons in the polymer backbone (e.g., 4, 6, 10 carbons) (Figure 4a). These reactions resulted in polymers with varied starting molecular weight but similar degradation profiles (Figure 4b).^98^ These PBAEs (PBAE‐4, PBAE‐6, and PBAE‐10) were degraded for defined times to form distinct molecular weight ranges, then incubated with DCs in either a soluble or particulate form. In soluble form, none of the PBAE formulations activated DCs.^98^ However, when condensed into a particulate form, PBAEs activated DCs in a molecular weight specific manner. PBAE‐4 particles activated DCs at molecular weights corresponding to early stages of degradation (less degradation time). Meanwhile, PBAE‐6 particles activated DCs after intermediate degradation times and PBAE‐10 showed little to no immunomodulatory activity, with minimal activation occurring only after long degradation times.^98^ Together, these results indicated that irrespective of degradation stage, the greatest activation levels were induced by particles formed from PBAEs fragments with molecular weights between 1,500 and 3,000 Da (Figure 4c).^98^ When DCs were treated with particles in the presence of an antigen, then co‐cultured with T cells, both antigen presentation and T cell proliferation increased signficantly.^97^ Similarly, when injected into lymph nodes of mice using a new technique to control the dose and combination of signals,^97^, ^99^, ^100^ immunogenic PBAE particles (“Particle”) activated lymph node resident innate immune cells compared to a sham injection (“Vehicle”), or an injection of soluble PBAEs (“Free”) (Figure 4d).^97^ These studies highlight the fact that materials may be non‐immunogenic or pro‐inflammatory at one time point, but as the material degrades, the physicochemical properties can change in ways that alter the immunogenicity. Analogous experiments are still necessary with other common polymers (e.g., PLGA) or extracellular matrix components (e.g., collagen, hyaluronic acid, and fibronectin) that cause differential immune responses as the scaffold degrades or is reabsorbed after implantation. In the case of these bioresorbable scaffolds, it would be advantageous to choose a material whose immunogenic profile promotes a natural wound healing response, with the implanted construct first promoting inflammation and immune cell recruitment then, as the material degrades, shifting toward a regenerative microenvironment.

With the growing understanding of the intrinsic immunogenicity of materials, an important concept is the interconnected nature of these properties. For instance, changing the shape of a particle by stretching may also change the size.^71^, ^72^ Similarly changing a functional group on the surface of a material may also change the surface charge and hydrophobicity; any of these variations might change the immune signatures of the materials.^88^ Therefore, one goal of future research should be to understand the relative contributions of properties or sets of properties to modulate immune function. An alternate strategy to understanding these interactions is to mimic the attractive features of synthetic materials, but eliminate carriers completely by self‐assembling immune signals into nanostructures.^101^, ^102^, ^103^, ^104^, ^105^, ^106^, ^107^, ^108^, ^109^ However, all of these strategies share the same goal, to design materials—whether natural or synthetic—that better control immune function.

## 4 TISSUE ENGINEERING CONSTRUCTS EXHIBIT INTRINSIC IMMUNOGENICITY

Classical tissue engineering approaches generally consist of scaffolds incorporating signals or cells that upon implantation, induce proliferation of the encapsulated cells, alter the phenotype of native cells infiltrating the implant, or promote changes in tissue growth and function. Mesenchymal stem cells are an immunomodulatory cell type that has been particularly important in this area to enhance responses to tissue engineering constructs.^110^, ^111^, ^112^, ^113^, ^114^, ^115^ These cells have the ability to differentiate into a number of cell lineages, making them attractive in tissue engineering and regenerative medicine approaches aimed at a range of tissue types.^110^, ^111^, ^112^, ^113^ Additionally, mesenchymal stem cells have the ability to interact with innate and adaptive immune cells, for instance, by secreting immune suppressive molecules such as prostaglandin E_2_.^110^, ^111^, ^112^, ^113^ Integration of chemical signals, growth factors, and cytokines into scaffolds is another important component widely explored in the field.^7^, ^39^, ^42^, ^43^ While these cells and signals incorporated in scaffolds and implants are being intensely studied, there is a gap in the understanding of how intrinsic immunogenicity of materials might be exploited to help tune and improve outcomes in tissue engineering. Building on the discussion above of what has been learned about inherent immunogenicity from the vaccine and immunotherapy area, this section will focus on how the physicochemical properties of common tissue engineering materials and extracellular matrix components can modulate immunological responses.

### 4.1 Macrophage responses to injury are influenced by the presence of acellular biomaterial scaffolds

Most tissue engineering approaches involve implants made from metallic, ceramic, or composite materials, or scaffolds comprised of synthetic materials, decellularized constructs, or extracellular matrix components.^15^ These structures must be able to overcome immunological rejection, a process resulting from recognition by innate and adaptive immune cells.^7^, ^25^ As explained in Section 2, macrophages play a large role in tissue repair with a balance of M1 and M2 phenotypes needed to promote proper healing. Interestingly, new studies show that the activation state of macrophages at the time of encounter with a biomaterial impact the uptake of the biomaterial and subsequent macrophage activation.^116^ For example, in the case of an injury where the wound is too large for conventional wound healing to occur, recovery would generally result in scar tissue formation. To test if addition of an acellular biomaterial scaffold could reduce adverse effects and promote proper healing, one recent report fabricated a scaffold by isolating extra cellular matrix components from a portion of the small intestine. This scaffold was then implanted at the injury site.^117^ In this study, the macrophage response to an untreated injury resulted in a pro‐inflammatory M1 phenotype, while implantation of the scaffold shifted this response to the M2 phenotype.^117^ This example highlights how just the presence of a biomaterial at an injury site can shift the immune response to promote tissue repair, motivating research to tease out the mechanisms that modulate immune function to promote healing.

### 4.2 Scaffold composition alters interactions with the immune system

**Figure 5:**
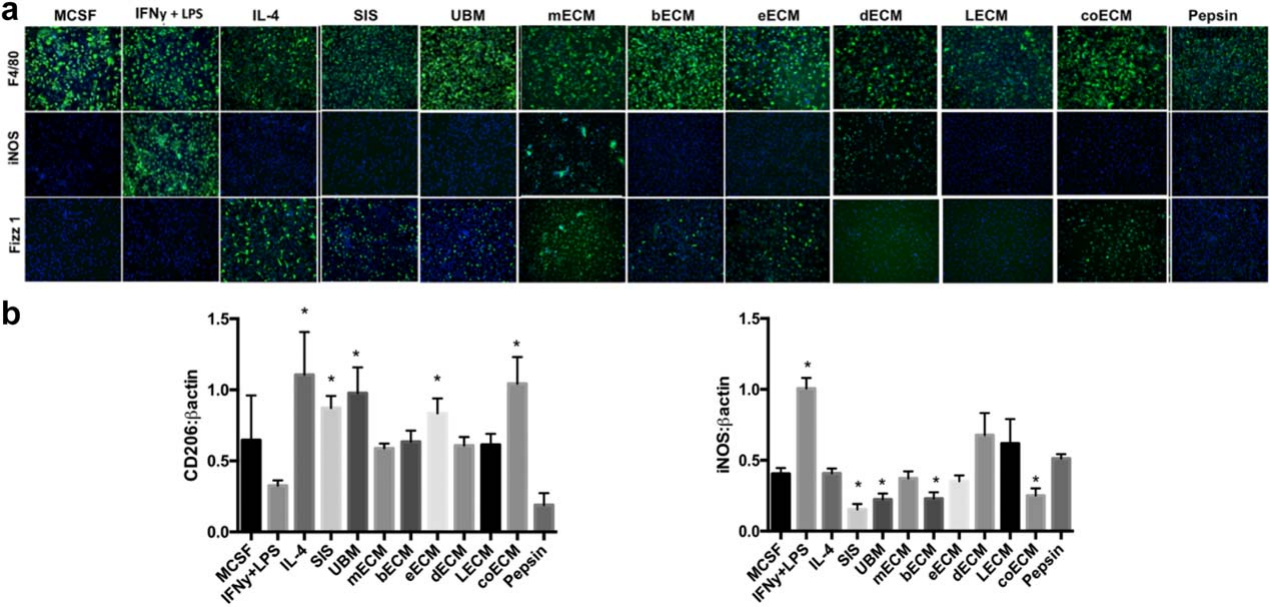
Scaffolds derived from extracellular matrix components of specific tissues polarize macrophage function. (a) Macrophages (stained with F4/80) and treated for 18 hr with control cytokines (MCSF, IFN‐γ + LPS, IL‐4) or solubilized extracellular matrix scaffolds cause differential expression of markers for the pro‐inflammatory M1 macrophage (iNOS) and the wound‐healing M2 macrophage (Fizz1) phenotypes. Solubilized scaffolds were produced via pepsin incubation of small intestine submucosa (SIS), urinary bladder (UBM), skeletal muscle (mECM), brain (bECM), esophagus (eECM), skin (dECM), liver (LECM), or colon (coECM) extracellular matrix components. MCSF was used as a negative control for macrophages while pepsin was a control for ECM solubilization, IFN‐γ + LPS was a positive control for M1 macrophages, and IL‐4 was a positive control for M2 macrophages. (b) Treatment with matrices also induces varied levels of M2 associated (CD206) and M1 associated (iNOS) proteins in macrophage lysates. Adapted with permission from Dziki JL, Wang DS, Pineda C, Sicari BM, Rausch T, Badylak SF. Solubilized extracellular matrix bioscaffolds derived from diverse source tissues differentially influence macrophage phenotype. *J Biomed Mater Res*. 2017;105(1):138‐147

There are a variety of biomaterials that can be used as tissue engineering constructs. Early research from the Babensee Lab highlights how DCs incubated on thin films of naturally occurring or synthetic materials—including PLGA, chitosan, alginate, hyaluronic acid, and agarose—cause differences in DC activation, cytokine production, and T cell proliferation.^26^, ^75^, ^118^ Based on these findings, recent studies have directly explored how materials commonly used in tissue engineering impact innate immune cells. In one study, extracellular matrix scaffolds were derived from diverse tissue sources including matrices from small intestine submucosa (SIS), urinary bladder (UBM), skeletal muscle (mECM), brain (bECM), esophagus (eECM), skin (dECM), liver (LECM), and colon (coECM) and enzymatically solubilized with pepsin.^119^ These solubilized extracellular matrix scaffolds were then used to treat macrophages. The level of M1 and M2 phenotype were determined by assessing iNOS and Fizz1 expression as markers for the M1 and M2 phenotypes, respectively.^119^ SIS, bECM, eECM, and coECM treatments induced an M2 phenotype at levels comparable to macrophages treated with a positive control signal (IL‐4) used to promote M2 differentiation (Figure 5a).^119^ In contrast, dECM treatment promoted a shift toward M1, with heightened iNOS expression on the macrophages stained with a classical macrophage marker, F4/80 (Figure 5a).^119^ Further investigation of macrophage lysates following treatment revealed SIS, UBM, bECM, and coECM solubilized ECM scaffolds caused decreased iNOS expression, while SIS, UBM, eECM, coECM increased CD206 expression; CD206 is another marker for the M2 macrophage phenotype (Figure 5b). These results confirmed a wound healing phenotype could be induced, and emphasizes that the selection of the tissue from which a scaffold is derived from can significantly bias immune function to improve scaffold performance.^119^

While the studies above used scaffolds prepared directly from complex tissues, other recent studies are investigating constructs formed from specific biological molecules or synthetic materials.^120^, ^121^, ^122^, ^123^, ^124^ One such molecule is collagen, a protein abundant in the extracellular matrix that has been widely explored as a scaffold component.^125^ During recent studies in mice, a collagen scaffold with silica integrated between the collagen fibers activated monocytes. These cells have the ability to differentiate into macrophages. Compared to sham surgeries, the scaffolds with silica promoted new bone and blood vessel formation.^120^ The authors hypothesized this response resulted from increased IL‐4 detected in serum one week after implantation; IL‐4 has previously been shown to induce wound healing phenotypes.^120^ Fibrin and fibrinogen have also been frequently used for tissue engineering applications; both of these proteins play a crucial role in the formation of blood clots during normal response to injury.^7^, ^15^, ^16^, ^17^ One study investigating the effect of fibrin and fibrinogen found that thin films of fibrin promoted macrophage secretion of anti‐inflammatory IL‐10, and were able to reverse the secretion of inflammatory cytokines (e.g., IFN‐γ) from macrophages stimulated with a TLR4 agonist.^121^ In contrast, soluble fibrinogen increased the pro‐inflammatory cytokine, TNF‐α. Interestingly, when macrophages were co‐treated with both fibrin films and soluble fibrinogen, fibrin played a dominate role, resulting in an anti‐inflammatory response.^121^ In a separate study, fibrinogen was prepared as a porous scaffold and the immunomodulatory effects on immune cells were investigated during a bone injury model. After implantation in mice, the fibrinogen scaffold outperformed sham implantations, promoting bone regrowth. These improvements were correlated with altered cytokine gene expression and changes in both the local and systemic immune cell responses.^122^ Similarly, other studies have investigated synthetic ceramic materials. These studies have revealed that treatment with a clinical standard for ceramic implants (tricalcium phosphate–hydroxyapatite) promotes an M1 macrophage phenotype.^123^ Alternatively, treatment with scaffolds formed from newer ceramic materials (e.g., baghdadite and strontium–hardystonite–gahnite) induced greater expression of markers indicative of M2 macrophage phenotypes.^123^ Last, synthetic polymers have also played a large role as scaffold materials. One recent example demonstrates that nondegradable polypropylene meshes induced M1 macrophages, while meshes coated with dermis or urinary bladder extracellular matrix components promote M2 macrophages.^124^ After implantation in rats, the meshes coated with extracellular matrix components also decreased the number of foreign body cells, a signal that a fibrous capsule would not form around the implant.^124^ These studies highlight how even with commonly used polymers or extracellular matrix components, dramatically different responses are possible because of differences in interactions with immune cells. However, further studies are needed to ascertain which properties of the scaffold are ultimately responsible for the immunogenic responses. With continued investigation into the chemical and biological differences between these ECM matrices and synthetic scaffolds, one could identify the key signals needed to shift the immune response toward a wound healing phenotype. After ascertaining which cues are important, future tissue engineering constructs could be designed with either synthetic materials supplemented with the pro‐regenerative signals or from a subset of the tissue ECM scaffolds that only contains the desired signals. This approach would allow for the fine tuning of the immune system response to the implanted biomaterial and promote tissue regeneration and wound healing.

### 4.3 Physicochemical properties of tissue engineering constructs also alter intrinsic immunogenicity

As with particle based delivery systems, the physicochemical properties of materials used in scaffolds can dramatically impact the interactions with the immune system. While this vein of research has garnered interest for particulate systems (see Section 3), interest in the tissue engineering field is just arising over the past few years. New studies are investigating how shape, composition, and charge of tissue engineering constructs impact the immune response.^126^, ^127^, ^128^, ^129^, ^130^, ^131^ For example, polymeric scaffolds have been synthesized by electrospinning polycaprolactone, then the resulting fibers were modified to exhibit different shapes.^126^ Scaffolds were then formed from either the random or aligned fibers. Afterward, these scaffolds were left unmodified or expanded to exhibit macro‐scale thicknesses of 3 or 10 mm.^126^ Following subcutaneous implantation into rats, macrophages were able to infiltrate into scaffolds formed from randomly aligned fibers with expanded thickness of 3 or 10 mm. However, scaffolds formed from aligned fibers that were expanded to 3 mm supported greater macrophage penetration and the smallest number of giant cells, a trait the authors attributed to the gap distance between the aligned fibers.^126^ In a similar study, scaffolds with varying pore sizes were formed from electrospun polydioxanone. These experiments revealed a correlation between increased pore size and a shift toward M2 function and away from M1 macrophages.^127^ An ongoing question in the tissue engineering field centers on the balance between the porosity of scaffolds and the mechanical properties required for the implant. As shown above, with increased porosity and an expanded conformation, tissue engineering constructs can promote pro‐regenerative environments by altering macrophage function. However, this change in scaffold structure may negatively influence mechanical strength and, in the case of tissue‐mimicking implants for structural components (e.g., bone), the mechanical strength of the scaffold needs to recapitulate that of the native tissue. Thus, while scaffold shape and porosity can be exploited to promote inflammation or repair by modulating macrophage phenotype and limiting fibrosis (i.e., reducing the number of foreign body cells), there is also a need to better understand the interplay between these immunological outcomes and material properties.^126^, ^127^

Another new avenue of research has focused on the chemical composition of scaffolds and how this influences the functions of DCs and macrophages. One study showed that calcium alginate gels promote inflammatory responses in cells and in mice by releasing calcium, a signal that increased DC activation and IL‐1β secretion.^128^ This work revealed the intrinsic immune function stemmed from the calcium, as gels formed with barium instead of calcium led to reduced scaffold immunogenicity.^128^ Since alginate hydrogels are commonly used for tissue engineering constructs and generally use calcium as the divalent ion for crosslinking, this discovery provides evidence that even the choice of molecule for cross linking could impact the resulting immune response and thus inform which divalent molecules should be used for crosslinking hydrogels. Experiments with hyaluronic acid formed into films via electrostatic interactions with poly‐l‐lysine caused monocytes to secrete pro‐inflammatory cytokines (e.g., IL‐1β, TNF‐α).^129^ Interestingly, after crosslinking the hyaluronic acid with aldehyde, there was a reduction in TNF‐α and IL‐1β secretion and slight increases in anti‐inflammatory cytokines.^129^ This result is in agreement with the studies discussed in Section 3.4, revealing increased activation from low molecular weight hyaluronic acid fragments; these particles might be generated as non‐crosslinked scaffolds begin to degrade. However, using crosslinking, the rate of degradation and the production of low molecular weight hyaluronic acid fragments generated via degradation could be slowed. This reduction decreased the overall level of pro‐inflammatory molecules. Other work investigated how the charge of hydrogel scaffolds impacts immunogenicity by evading foreign‐body reactions.^130^ In this study, zwitterionic poly(carboxybetaine methacrylate) hydrogels prepared from a carboxybetaine monomer and a carboxybetaine cross‐linker shifted macrophage phenotype to an anti‐inflammatory state compared to samples treated with poly(2‐hydroxyethyl methacrylate) hydrogels, a commonly used molecule similar to PEG that reduces protein absorption.^130^ Thus, the hydrogels were able to diminished macrophage activation in mice by reducing the underlying protein adsorption to the scaffold after implantation.^130^ This feature could be beneficial for future implanted biomaterial scaffolds designed to limit fibrotic buildup and implant rejection. Together, the results from this section suggest that the material with which a hydrogel is formed, the extent of crosslinking, and the molecule used for crosslinking can all impact the immunogenicity of the scaffold. Similar to the discussion in Section 3.4, there is an ongoing need to understand the interplay between these properties. For instance, if a hydrogel is formed from a material that is immunostimulatory as it degrades, crosslinking of this scaffold with calcium may result in reduced immunogenicity by reducing the degradation but could also cause a local increase in pro‐inflammatory cytokines. Therefore, it will be crucial for future tissue engineering approaches to understand which properties of hydrogels are the main drivers of this intrinsic immunogenicity.

**Figure 6:**
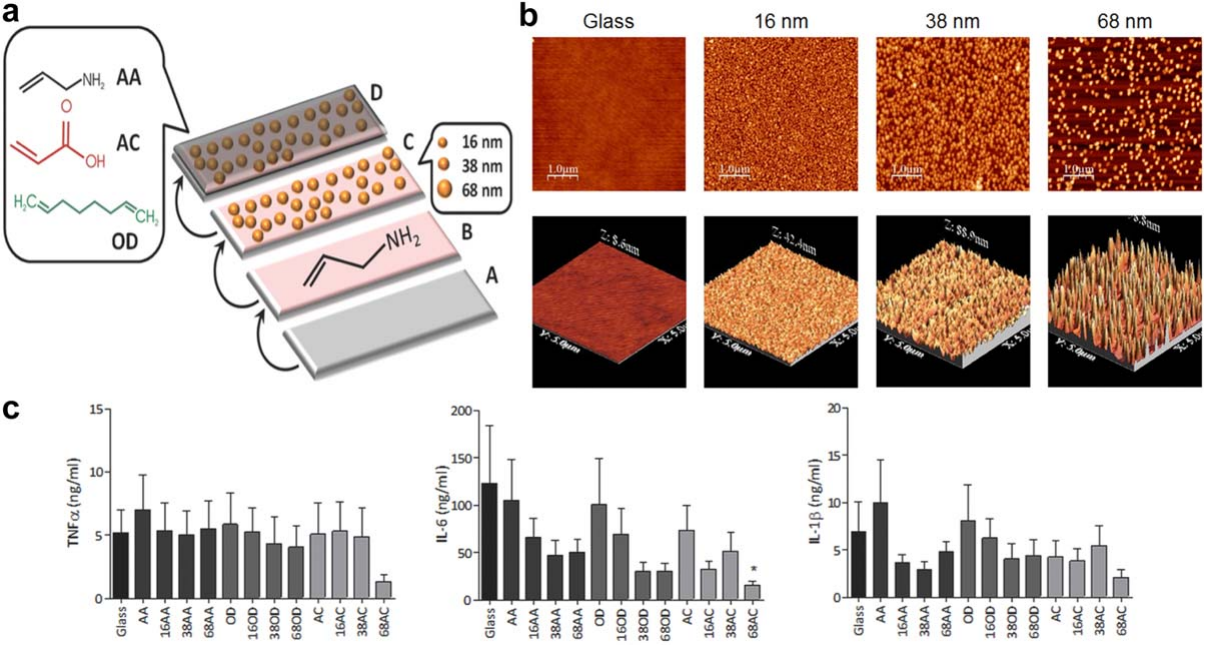
Implant surface morphology and chemical composition induces innate cell activation and cytokine secretion. (a) Schematic depiction of glass cover slips coated with gold nanoparticles, then functionalized with allylamine (AA), octadiene (OD), or acrylic acid (AC) to form biomimetic surfaces abundant in amino, alkyl, or carboxylic acid groups, respectively. (b) Atomic force micrographs showing 2‐D (top) and 3‐D (bottom) surfaces with different roughness due to the different particles diameters. (Top scale bar, 1 µm; lower scale 5 µm × 5 µm). (c) Macrophages cultured on these surfaces exhibited different secretion levels of pro‐inflammatory cytokines TNF‐α (left), IL‐6 (center), and IL‐1β (right); cells cultures on the roughest surfaces (prepared with 68 nm particles) reduced inflammatory cytokine secretion. Reprinted with permission from Christo SN, Bachhuka A, Diener KR, Mierczynska A, Hayball JD, Vasilev K. The role of surface nanotopography and chemistry on primary neutrophil and macrophage cellular responses. *Adv Healthcare Mater*. 2016;5(8):956‐965

In an elegant study by Christo et al., complex surface nanotopography and chemical composition commonly seen in tissue engineering constructs were mimicked to test the impact on the response of innate immune cells.^131^ Glass cover slips were coated with gold nanoparticles exhibiting varying diameters. These materials were then modified by deposition of allylamine (AA), octadiene (OD), or acrylic acid (AC) to create surfaces abundant in either amino, alkyl, or carboxylic acid groups, respectively (Figure 6a).^131^ Because the surfaces were formed with nanoparticles of varying diameters, these constructs mimicked scaffolds with either smooth surfaces or exhibited tunable degrees of surface roughness (Figure 6b).^131^ When neutrophils were incubated with the surfaces, rough and, in particular, acidic surfaces increased section of MMP‐9, a protein involved in extracellular matrix degradation.^131^ Additionally, when incubated with macrophages, these surfaces caused differential effects on the secretion of pro‐inflammatory cytokines. The rough, acidic surfaces again shifted the environment away from inflammation by decreasing the secretion of pro‐inflammatory cytokines TNF‐α, IL‐6, and IL‐1β (Figure 6c).^131^ This type of experiment, where multiple parameters are systematically investigated, could inform the rational design of future scaffolds fabricated with physicochemical properties that can modulate the immune response between inflammation and tissue repair.

## 5 NEW TECHNOLOGIES AND ANALYSIS METHODS WILL EXPLOIT INTRINSIC IMMUNOGENICITY TO ADVANCE TISSUE ENGINEERING CAPABILITIES

**Figure 7:**
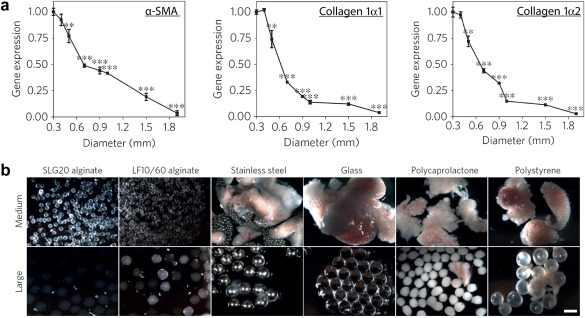
Implanted materials with distinct sizes and compositions alter fibrotic capsule formation. (a) Following implantation of alginate spheres of different sizes into the peritoneum of mice, gene expression profiles of the pro‐fibrotic markers α‐SMA (left), collagen 1α1 (center), and collagen 1α2 (right) revealed larger particles reduced fibrotic build‐up. (b) Images revealing the level of fibrosis for particles with diameters of 0.5 mm (medium) or 1.5–2 mm (large) prepared from alginate, stainless steel, glass, polycaprolactone, or polystyrene. For all materials, large particles were associated with reduced fibrosis 14 days after implantation. (Scale bar, 2 mm). Adapted with permission from Macmillan Publisher Ltd: *Nature Materials* from Veiseh O, Doloff JC, Ma M, et al. Size‐ and shape‐dependent foreign body immune response to materials implanted in rodents and non‐human primates. *Nat Mater*. 2015;14(6):643‐651, copyright 2015

Recent studies in tissue engineering investigating the impact of material properties on immune activation have yet to fully elucidate the mechanism through which this activation occurs. Future research endeavors will need to incorporate more experimental parameters and higher, more complex animal models to determine the biomaterial features that contribute the most to immunogenicity. In one such study from the Langer and Anderson labs, mouse, rat, and non‐human primate models were used to investigate how the size and shape of various biomaterial implants fabricated from ceramics, hydrogels, metals, and plastics activated innate cells and led to fibrosis.^132^ First, alginate particles were prepared with sizes from 0.3 to 1.9 mm, then implanted in mice.^132^ Following implant retrieval after 2 weeks, particles size was inversely correlated with expression of genes involved in fibrosis: α‐SMA (Figure 7a, left), collagen 1α1 (Figure 7a, center), and collagen 1α2 (Figure 7a, right).^132^ After determining that size impacted the foreign body response to alginate, the authors compared the response to different biomaterials exhibiting similar sizes. Particles with a diameter of 0.5 mm were formulated from alginate, stainless steel, glass, polycaprolactone, or polystyrene, then implanted intraperitoneally in mice. The implants were removed after 14 days, and all resulted in fibrotic growth (Figure 7b, top).^132^ Interestingly, particles fabricated from the same materials but with larger diameters (1.5–2 mm) reduced fibrotic tissue formation (Figure 7b, bottom), a phenomena attributed to reduced infiltration of macrophages and neutrophils at the implant site.^132^ Further investigation with alginate particles of medium (0.5 mm) and large (1.5 mm) sizes implanted into non‐human primates resulted in similar findings.^132^ After implantation of both medium (0.5 mm) and large (1.5 mm) particles into mice, the number of neutrophils and macrophages on the excised particle as well as macrophage phenotype was determined at various times post‐implantation (e.g., day 1, 4, and 7).^132^ Larger particles caused a shift toward immune regulatory and wound healing macrophage phenotypes, while medium particles biased responses toward inflammatory phenotypes.^132^ In another study from the Anderson lab, the mechanism through which fibrosis occurred after implantation of alginate spheres was determined.^133^ This study incorporated both mouse and primate models to study the response to the implanted particles. While implantation of alginate spheres increased the macrophage, neutrophil, and B cell responses to the implants, macrophages and not neutrophils were determined to be the main cell type that drives fibrosis.^133^ Using knock out mice for neutrophils, macrophages, and B cells, it was determined that macrophages were essential for the formation of fibrosis on the alginate spheres and lead to B cell recruit which further promoted fibrosis.^133^ High throughput gene expression analysis revealed colony stimulating factor‐1 receptor was ultimately the driving mechanism for fibrosis in response and direct inhibition of this receptor was able to control fibrosis without the complete depletion of macrophage function.^133^ These studies highlight the utility of using multiple animal models and emerging technologies to determine how specific physicochemical properties influence immune responses to tissue engineering constructs. Such approaches could provide a generalizable framework for other materials and questions.

**Figure 8:**
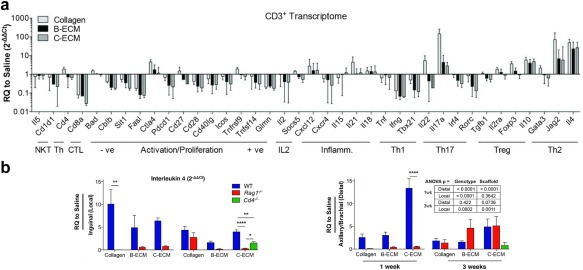
Adaptive immune cells play a role in the response to implanted scaffolds. After inducing a critical muscle injury in mice, scaffolds derived from collagen, bone (B‐ECM), or cardiac muscle extracellular matrix (C‐ECM) were implanted. (a) CD3^+^ T cell transcriptome analysis was used to evaluate the gene expression of markers for distinct immune cell populations and phenotypes compared to a sham saline surgery control, denoted as “RQ to Saline.” Treatment with B‐ECM and C‐ECM scaffolds revealed an increase in T_H_2‐associated genes (e.g., Jag2, IL‐4). (b) Treatment with scaffolds in wild‐type mice (blue, “WT”) increased IL‐4 gene expression in local (left) and distal lymph nodes (right) compared to saline treatments. When CD4^+^ T cell‐deficient mice (green, “Cd4^−/−^”), or B and T cell‐deficient mice (red, “Rag1^−/−^”) were treated with ECM scaffolds, IL‐4 gene expression decreased. (a and b) from Sadtler K, Estrellas K, Allen BW, et al. Developing a pro‐regenerative biomaterial scaffold microenvironment requires T helper 2 cells. *Science*. 2016;352(6283):366‐370. Reprinted with permission from AAAS

Other recent research has focused not just on innate immunity, but on the role of implanted materials in modulating adaptive immunity. In a study by the Elisseeff and Pardoll labs, scaffolds were derived from bone (B‐ECM) and cardiac muscle (C‐ECM) extracellular matrix components, then used to treat critical muscle injuries in mice.^134^ The response to these scaffolds was compared to collagen scaffolds and saline treatments to study the underlying immune mechanisms.^134^ In a normal immune response to a pathogen, APCs such as macrophages and DCs present antigen to naïve, CD3^+^ T cells which then proliferate and, depending on the signals also delivered to the T cell, differentiate into various T cell subsets.^7^ These subsets consist of cytotoxic CD8^+^ T cells that kill infected host cells and CD4^+^ T cells that include T helper 1 (T_H_1) cells which assist in M1 macrophage activation, T helper 2 (T_H_2) cells that promote M2 macrophage activation, and T_H_17 T cells which cause inflammation through activation of neutrophils.^7^ In wild type mice, treatment with collagen, B‐ECM, and C‐ECM scaffolds increased CD4^+^ T cells and the expression of IL‐4 genes, an important cytokine for T_H_2 cell differentiation and tissue repair via M2 macrophages.^134^ Supporting this finding, transcriptome analysis of genetic material from CD3^+^ T cells one week after treatment with the biomaterial scaffolds revealed increased messenger RNA that drives development of T_H_2 responses (Figure 8a).^134^ When repeating these treatments in Rag1^−/−^ mice, which are deficient in mature B and T cells, IL‐4 gene expression was decreased to levels comparable to saline treatments, further supporting the hypothesis that T cells were responsible for IL‐4 production.^134^ Additional studies showed the increase in IL‐4 expression was present in both the draining (e.g., inguinal) and distal lymph nodes (e.g., axillary, brachial) (Figure 8b), indicating local changes to immune function can lead to systemic responses.^134^ When mice deficient in CD4^+^ T cells were used, this response was diminished, although not as low as measured when both B and T cells were knocked out in Rag1^−/−^ mice (Figure 8b).^134^ These results suggest T_H_2 cells play a critical role in wound healing and creating a regenerative microenvironment, while other adaptive immune cells (e.g., CD8^+^ T cells, B cells) may play a supporting role in these processes.^134^ While the majority of research in tissue engineering has focused on the innate immune cells (e.g., macrophages) that play a major role in both regeneration and in scaffold failure, this study reveals the need for more work elucidating the role that adaptive immune cells play in promoting or inhibiting tissue repair. Through a better understanding of how T cells support tissue regeneration or how antibodies produced by B cells impact a host reaction to implanted materials, future constructs could be designed with materials that control these pathways.

As the field of tissue engineering and regenerative medicine progresses, novel biomaterials and scaffold preparation methods will motivate further studies to investigate the immunogenicity of these new materials. One exciting avenue of research is 3‐D printing of tissue engineering constructs.^135^, ^136^, ^137^, ^138^ These new approaches allow for the synthesis of implants with precise shapes and architectures fabricated from a variety of materials. In a recent study from Kang et al., biodegradable polymers and hydrogels encapsulating cells were printed together in human‐scale and tissue shape‐mimicking constructs.^139^ This system allowed mixing of polycaprolactone with hydrogels consisting of gelatin, fibrinogen, hyaluronic acid, and glycerol to create stable structures with various pore sizes.^139^ Scaffolds prepared in this way supported high viability of implanted cells and promoted tissue reconstruction for a variety of tissues types, including bone, cartilage, and muscle.^139^ While this research presents a new, flexible system to synthetically create biomaterial‐based scaffolds, the researchers note that the host immune response to their new materials was not investigated.^139^ This comment highlights some of the broader issues facing the field, in particular, that systematic studies will be important to fully understand the mechanisms through which the immune system is activated and interacts with these constructs during wound healing and regenerative processes.

## 6 CONCLUSION

The vaccine and immunotherapy fields have provided valuable insight into how material properties impact immune responses. This work has already shown physicochemical features alter the immunogenicity of biomaterials and is helping to inform the design of better materials that actively drive immune response toward a desired outcome. The field of tissue engineering provides a rich avenue to explore some of these same concepts and opportunities. With new knowledge of how these physicochemical properties influence the response to implanted materials, greater flexibility will arise for controlling immune response by carefully designing or selecting the material properties of implants and scaffolds. To reach this goal, future studies should broaden in vitro research to understand how well‐controlled, 3D architecture might impact intrinsic immunogenicity, and utilize animal models where material constructs with systematically introduced property variations are studied. Conducting these studies in the absence, and in the presence, of other immunomodulatory factors will help reveal the interplay between material properties and the resulting local and systemic immune responses.
